# The Association between Nonylphenols and Sexual Hormones Levels among Pregnant Women: A Cohort Study in Taiwan

**DOI:** 10.1371/journal.pone.0104245

**Published:** 2014-08-22

**Authors:** Chia-Huang Chang, Ming-Song Tsai, Ching-Ling Lin, Jia-Woei Hou, Tzu-Hao Wang, Yen-An Tsai, Kai-Wei Liao, I-Fang Mao, Mei-Lien Chen

**Affiliations:** 1 Institute of Environmental and Occupational Health Sciences, School of Medicine, National Yang Ming University, Taipei, Taiwan; 2 Department of OBS & GYN, Cathay General Hospital, Taipei, Taiwan; 3 School of Medicine, Fu Jen Catholic University, Taipei, Taiwan; 4 Department of Endocrinology and Metabolism, Cathay General Hospital, Taipei, Taiwan; 5 Department of Pediatrics, Cathay General Hospital, Taipei, Taiwan; 6 Department of Obstetrics and Gynecology, College of Medicine, Chang Gung University Genomic Medicine Research Core Laboratory (GMRCL), Chang Gung Memorial Hospital, Chang Gung Memorial Hospital, Taoyuan, Taiwan; 7 Department of Occupational Safety and Health, Chung Shan Medical University, Taichung, Taiwan; Kagoshima University Graduate School of Medical and Dental Sciences, Japan

## Abstract

**Background:**

Nonylphenol (NP) has been proven as an endocrine disrupter and had the ability to interfere with the endocrine system. Though the health effects of NP on pregnant women and their fetuses are sustained, these negative associations related to the mechanisms of regulation for estrogen during pregnancy need to be further clarified. The objective of this study is to explore the association between maternal NP and hormonal levels, such as estradiol, testosterone, luteinizing hormone (LH) and follicle stimulating hormone (FSH), and progesterone.

**Methods:**

A pregnant women cohort was established in North Taiwan between March and December 2010. Maternal urine and blood samples from the first, second, and third trimesters of gestation were collected. Urinary NP concentration was measured by high-performance liquid chromatography coupled with fluorescent detection. A mixed-effects model using a generalised estimating equation (GEE) was applied to assess the associations between maternal NP concentration and plasma hormones throughout the three trimesters.

**Results:**

In total, 162 singleton pregnant women completed this study through delivery. The geometric mean of creatinine-adjusted urinary NP concentrations were 4.27, 4.21, and 4.10 µg/g cre. in the first, second, and third trimesters respectively. A natural log-transformation of urinary NP concentrations were significantly associated with LH in the GEE model (β = −0.23 mIU/ml, p<0.01).

**Conclusion:**

This perspective cohort study demonstrates that negative association occurs between maternal NP exposure and plasma LH levels. The estrogen-mimic effect of NP might influence the negative feedback on LH during pregnancy.

## Introduction

Nonylphenol (NP), a member of alkylphenols is used in industrial processes including the manufacture of polyvinyl chloride products, medical products, cosmetics, children's toys, cleaning supplies, and plastic additives [1], [2], [3], [4]. Nonylphenol ethoxylates (NPEs), the precursors of NP, which have both hydrophilic and hydrophobic properties are nonionic surfactants and used as detergents, emulsifiers, and numerous other products in household and agricultural applications. NPEs in the environment biodegrade to shorter-chain derivatives and subsequently to NP. Due to the intensive use of NP, the annual production of NP is substantial [3]. Widespread human NP exposure occurs mainly through the ingestion of NP-contaminated water and foods [1], [5], [6], [7], [8].

It has been proven that NP can affect the normal function of the endocrine system by interacting with estrogen receptors [4], [9], [10], [11]. As an estrogen-mimic, NP had the ability to compete with estradiol or promegestone for estrogen and progesterone receptor binding [4], [10]. Reproductive toxicities were indicated among numerous species exposed to NP [12], [13], [14], [15], [16], [17], [18], [19], [20]. These adverse effects included lesions of gonadal development, testicular abnormalities, inhibition of ovarian development, and reduction in the reproductive organ weights.

In Taiwan, NP has been detected in most rivers, sludge, and sediments [21], [22], [23], [24], [25]. The unrestricted and intensive use of NPEs in detergents for daily activities may result in the high and ubiquitous levels of NP in Taiwan. Previous study reported that the average daily intake of NP for Taiwanese individuals (28.0 µg/day) was high in comparison with that in Germany (7.5 µg/day) and New Zealand (alkylphenol: 3.0–3.6 µg/day) [6], [7], [8]. In addition, high NP exposure levels among those susceptible and vulnerable subjects, such as fetuses, adolescents, and pregnant women were also determined [26], [27], [28]. The health effects of high internal NP levels need to be concerned.

Our previous cohort study demonstrated that maternal NP exposure is associated with small for gestational age, decreased body length at birth, and low maternal weight gain [27], [29]. Though the xeno-estrogenic effects of NP on pregnant women and their fetuses are sustained, these negative associations related to the mechanisms of regulation for estrogen during pregnancy need to be further clarified. The objective of this study is to explore the association between maternal NP and hormones including estradiol (E2), testosterone, luteinizing hormone (LH) and follicle stimulating hormone (FSH), and progesterone.

## Materials and Methods

### 2.1 Study design and subject recruitment

The pregnant cohort in North Taiwan was identical to those in the previous study [27], [29]. The research protocol was approved by the Institutional Review Board (IRB) of Cathay General Hospital in Taipei. Pregnant women who underwent an ultrasonic scan during the first trimester at an obstetrics clinic were invited to participate in this cohort study. The inclusion criterion was set that the fetal heart beat was detected at the first prenatal visit; fetuses with structural abnormalities or chromosomal defects were set as an exclusion criterion. In total, 235 pregnant women were invited for convenient sampling, and 201 women agreed to participate this study. The response rate was 85.5%.

An informed consent form was used to obtain written consent from all participants. After written consent forms were signed, face-to-face interviews were conducted to collect information on their socio-demographic characteristics. At the time of recruitment, each woman was asked to complete a structured questionnaire requesting information about lifestyle (*i.e.*, stress, frequency of using detergent and plastic products, consumption of healthy food and medication) and dietary consumption (*i.e.*, meat, vegetable, fruit, tea, and coffee consumption). Pregnant women were asked about the frequency (times per day/week/month) and portions (small/medium/large) ingested using photographs of example foods. The questionnaire was administered in the first trimester and again in the third trimester to identify changes in lifestyle and dietary intake during pregnancy.

### 2.2 Specimens collection

Maternal urine and blood samples of the first, second, and third trimester were collected during the routine examinations: at Down's syndrome screening during the first trimester, gestational weeks 10–13, gestational diabetes mellitus screening during gestational weeks 24–28, and admission for delivery in the third trimester. A spot urine was collected in a 30 ml brown glass vessel. Maternal blood sample was drawn in a 10 mL glass Vacutainer with K_2_EDTA. Plasma was fractioned by centrifugation at 3000 rpm for 10 min. After collection, all samples were stored at −80°C until analysis. Urine samples were analysed for NP and creatinine.

### 2.3 NP analysis

The analytical method was identical to the previous study [30]. The pHs of all urine samples were adjusted to a level of 5.5 by adding 1 M acetic acid (Merck, Germany) and mixing with 1 mL of 1 M ammonium acetate solution (Merck, Germany). Specimens were deconjugated using β-glucuronidase (Sigma-Aldrich, USA), incubated for 15 h at 37°C in a shaker bath, and acidified to a pH of 3 using 1.0 M hydrochloric acid (Merck, Germany). Deconjugated samples were cleaned with pH solid-phase extraction (SPE) cartridges (Supelco, USA). The SPE cartridge was preconditioned with 20 mL of methanol followed by 3 mL of pure water. After sample application, the cartridge was washed with 5 mL of pure water. Finally, the analytes were eluted with 3 mL of methanol and were determined using high-performance liquid chromatography coupled with fluorescent detection (Hitachi, Tokyo). The reverse-phase column was a Luna C18 (250×4.6 mm) with a 5-µm particle size (Phenomenex, USA). The isocratic mobile phase was a mixture of acetonitrile∶water (75∶25, v/v) with a flow rate of 1.0 ml/min. The fluorescent detector was operated with an excitation wavelength of 275 nm and emission wavelength of 300 nm. The samples were injected in quantities of 20 µL. All urinary NP concentrations were adjusted by creatinine.

### 2.4 E2 analysis

E2 was examined by enzyme-linked immunosorbent assay (ELISA) using Coat-A-Count Estradiol kit. Plasma (100 µL) was mixed with 1.0 ml of iodine-125 labeled estradiol and then incubated for 3 h at room temperature. Using a foam decanting rack to aspirate the contents of all tubes and drain for 2–3 minutes. The tube was counted using a gamma counter. The concentration of E2 was determined by comparing the counts to a calibration curve.

### 2.5 Testosterone and Progesterone Analysis

Testosterone and progesterone were examined by radioimmunoassay (RIA) using TESTO-CT2 RIA kit and PROG-CTRIA kit, respectively. Plasma (25 µL) was mixed with 500 µL of iodine-125 labeled testosterone (or progesterone) and incubated for 1 h at 37°C in a water bath. The liquid was aspirated, and each tube was rinsed with 1 ml of distilled water. The distilled water was aspirated immediately, and the remaining radioactivity bound to the tubes was measured using a gamma scintillation counter calibrated for iodine-125.

### 2.6 LH and FSH analysis

LH and FSH were examined by immunoradiometric assay (IRMA) using Coat-A-Count LH (or FSH) IRMA kit. Plasma (200 µL) was thoroughly mixed with 100 µL of iodinated anti-LH (or FSH) polyclonal goat antibody and shaked for 60 minutes on a rack shaker. The shaken plasma was decanted thoroughly, and 2 mL of Buffer Wash Solution was added. After waiting 1 to 2 minutes, the contents were decanted thoroughly and drained for 2 or 3 minutes. All residual droplets were shaken off, and the samples were counted for 1 minute using a gamma counter.

Quality control hormones samples were used in each series of assays to check the quality of the results obtained.

### 2.7 Statistical analysis

SAS version 8.1 was used for the statistical analysis. The distribution of maternal NP and sexual hormones in the three trimesters were presented in a box plot. The correlations among NP, E2, testosterone, LH, FSH, and progesterone were initially explored using Spearman's correlation. Potential confounders were considered based on maternal factors including age, height, weight, gestational age, and weight gain. Pregnant women with adverse pregnancy outcomes were also considered. A natural log-transformation of NP data was used in this study. Because of the longitudinal data, a mixed-effects model using a generalised estimating equation (GEE) was used to assess the associations between maternal NP and hormones concentrations in the three trimesters. Statistical significance was set at p<0.05.

## Results

**Figure 1:**
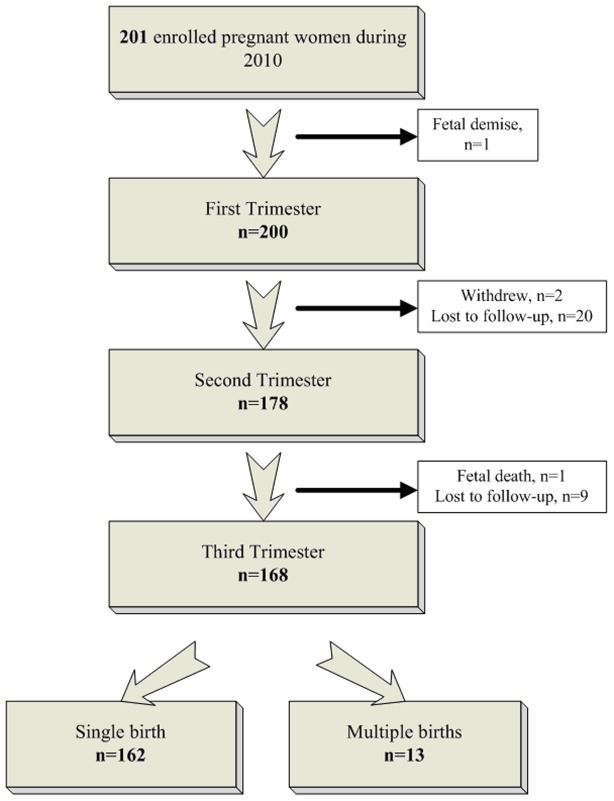
Follow-up of the cohort of pregnant women.

A total of 162 singleton pregnant women followed until delivery (Fig. 1). The average age of the pregnant women was 31 years old; 25% were at an advanced maternal age (defined as maternal age greater than 34 years in the first trimester). The average body mass index (BMI) was 21.2 kg/m^2^ before gestation and 26.3 kg/m^2^ at delivery respectively and maternal total weight gain during gestation was 13.0 kg. More than 10% (N = 19) of the pregnant women were classified as overweight or obese. Most pregnant women (114/162, 70.4%) had an education level of bachelor's degree or higher. Forty percent were primiparous. All pregnant women were not occupationally exposed to NP. For example, thirty-four percent of the pregnant women worked in the wholesale and retail sector, 28.4% were housewives and others (e.g., a part-time job or self-employed job in the wholesale and retail sector or owning a business.) (Table 1).

**Table 1:** The socio-demographic characteristics of singleton pregnant women.

	N (%)	Mean (SD)
Age (years)		31.7(4.4)
<30	52 (32.1)	
30–34	69 (42.6)	
>34	41 (25.3)	
Pre-pregnancy BMI (kg/m^2^)		21.2 (3.2)
<18.5	26 (16.1)	
18.5–25	117 (72.2)	
≧25	19 (11.7)	
Maternal BMI at delivery (kg/m^2^)^b^		26.3 (3.1)
18.5–24.9	66 (40.7)	
≧25	96 (59.3)	
Total weight gain (kg)		13.0 (4.3)
Parity		
Primiparous	63 (38.9)	
Multiparous	99 (61.1)	
Occupation		
Wholesale and retail	55 (34.0)	
Medical and health	15 (9.3)	
Service industry	21 (13.0)	
Housewife	46 (28.4)	
Others	25 (15.4)	

SD: standard deviation.

Before and during pregnancy, the proportion of nutriment supplementation increased from 45.5% to 86.1% and that of medication use decreased from 10.5% to 5.7%. No women smoked or drank alcohol during gestation. The dietary habits of pregnant women were assessed using a semi-quantitative pattern. Only the frequencies of nutrient supplementation and of whole milk significantly increased during gestation.

**Figure 2:**
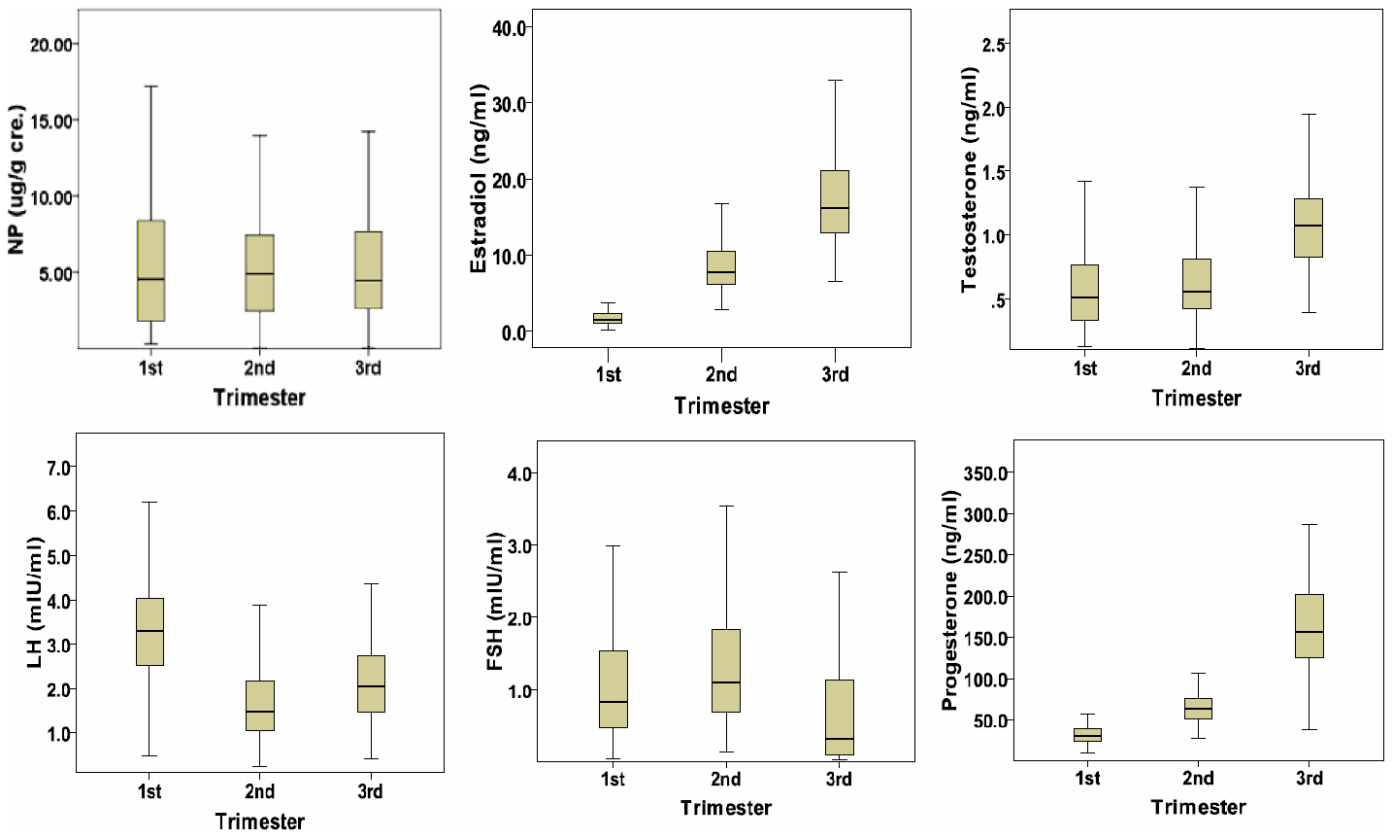
Urinary NP and plasma sexual hormones concentrations of pregnant women during the three trimesters.

Detection rates for urinary NP during the three trimesters were 100%, 99.4%, and 96.7%, respectively. After adjusting for urinary creatinine concentration, NP concentrations during the three trimesters were 4.27, 4.21, and 4.10 µg/g cre., respectively. The urinary NP concentration decreased stepwise with pregnancy progression, however, differences were not statistically significant. The E2, testosterone and progesterone increased with the progression of gestational trimesters (Fig. 2). The geometric mean during the three trimesters were 1.47, 7.72, and 16.45 ng/ml for E2, 0.50, 0.59, and 1.09 ng/ml for testosterone, 30.50, 62.92, and 155.73 ng/ml for progesterone, respectively (Table 2).

**Table 2:** Maternal urinary NP and plasma hormones concentrations during the three trimesters.

	1st trimester	2nd trimester	3rd trimester
	Geometric mean (range)		
NP (µg/g cre.)	4.27 (0.45–62.61)	4.21 (0.04–94.93)	4.10 (0.04–48.45)
Estradiol (ng/ml)	1.47 (0.19–3.79)	7.72 (2.80–17.68)	16.45 (6.58–36.38)
Testosterone (ng/ml)	0.50 (0.12–2.58)	0.59 (0.11–2.43)	1.09 (0.39–4.25)
LH (mIU/ml)	3.12 (0.49–9.04)	1.52 (0.24–6.45)	2.00 (0.43–10.32)
FSH (mIU/ml)	0.78 (0.06–4.69)	1.14 (0.15–6.58)	0.38 (0.04–6.55)
Progesterone (ng/ml)	30.50 (11.58–63.6)	62.92 (26.92–118.74)	155.73 (36.80–400.10)

**Figure 3:**
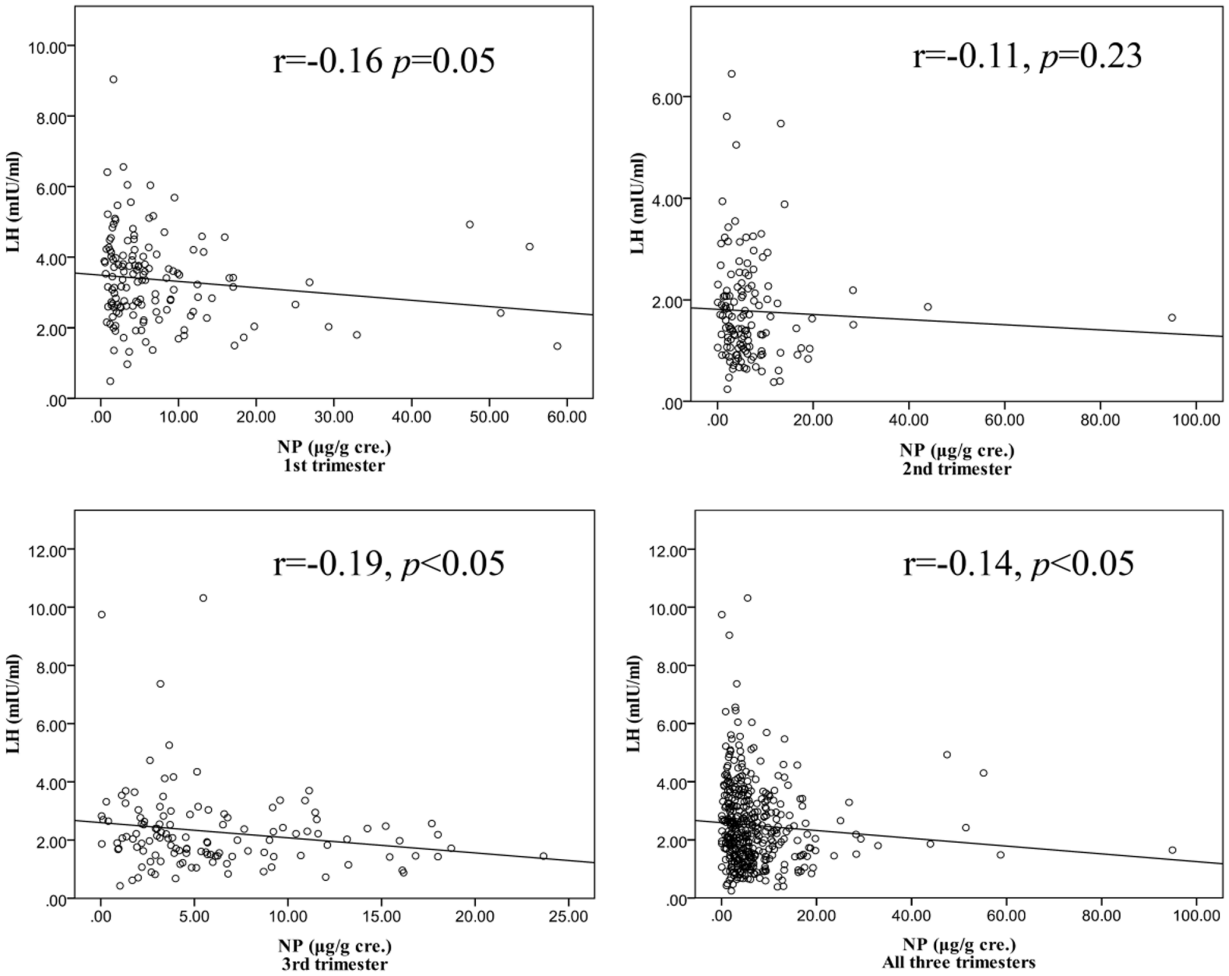
The scattered plot for maternal urinary NP and plasma LH levels in the three trimesters.

The significantly negative correlations existed between maternal NP levels and plasma LH (1st trimester, r = −0.16; 3rd trimester, r = −0.19) or FSH (1st trimester, r = −0.21) (Fig. 3 & Table 3). A GEE model used to determine whether maternal NP concentrations were associated with hormone concentrations throughout all three trimesters showed that there were significant correlations between urinary NP concentrations and LH (β = −0.02 mIU/ml, p value = 0.02). A natural log-transformation of urinary NP concentrations associated with the decrease in LH (β = −0.23, p value = 0.02). After adjusting other covariates, the plasma levels of E2, testosterone and progesterone still increased with the gestational age (Table 4).

**Table 3:** The Spearman's correlation between maternal urinary NP and plasma hormones levels.

	1st-trimester		2nd-trimester		3rd-trimester	
Variables	r	p-value	r	p-value	r	p-value
Estradiol	0.10	0.24	0.13	0.14	0.02	0.78
Testosterone	0.03	0.75	0.07	0.42	0.02	0.86
LH	−0.16	0.05	−0.11	0.23	−0.19	0.03
FSH	−0.21	0.01	0.09	0.30	0.04	0.64
Progesterone	0.11	0.20	0.14	0.10	−0.10	0.24

r = Spearman's correlation coefficient.

**Table 4:** Generalised estimating equation model for maternal NP and hormones levels throughout all three trimesters.

	Estradiol (ng/ml)		Testosterone (ng/dl)		LH (mIU/ml)		FSH (mIU/ml)		Progesterone (ng/ml)	
Variables	β (SE)	p-value	β (SE)	p-value	β (SE)	p-value	β (SE)	p-value	β (SE)	p-value
Age (years)	−0.01 (0.06)	0.97	−0.01 (0.01)	0.20	−0.01 (0.02)	0.77	0.01 (0.01)	0.67	−0.22 (0.37)	0.55
Gestational age (weeks)	0.57 (0.03)	<0.01	0.02 (0.01)	<0.01	−0.04 (0.01)	<0.01	−0.01 (0.01)	0.36	5.17 (0.22)	<0.01
BMI (kg/m^2^)	−0.17 (0.08)	0.04	0.03 (0.01)	0.03	−0.01 (0.02)	0.71	−0.02 (0.02)	0.21	0.61 (0.51)	0.23
Parity	1.27 (0.56)	0.02	0.12 (0.07)	0.09	0.11 (0.15)	0.49	0.18 (0.12)	0.13	−1.94 (3.55)	0.59
Birth sex	−0.67 (0.49)	0.17	−0.05 (0.06)	0.41	−0.08 (0.14)	0.59	−0.05 (0.10)	0.62	6.96 (3.13)	0.03
NP (µg/g cre.)	−0.02 (0.02)	0.25	−0.01 (0.01)	0.39	−0.02 (0.01)	0.02	0.01 (0.01)	0.26	0.07 (0.16)	0.64
NP^a^	−0.10 (0.31)	0.73	0.02 (0.02)	0.52	−0.23 (0.09)	<0.01	0.01 (0.04)	0.82	−0.39 (1.58)	0.80

a : Beta of a natural log-transformation of urinary NP concentrations after adjusting covariates including age, gestational age, BMI, parity, birth sex, and adverse pregnancy outcomes.

Parity: primiparas as a reference group.

Birth sex: female birth as a reference group.

β = estimated coefficient.

SE = standard error of the estimated coefficient.

## Discussion

### 4.1 Hormonal regulation for pregnancy

Hormonal regulation of pregnant women plays an important role in the process of fetal growth and development: it is critical for initiation and maintenance of pregnancy, promotes the expression of critical growth factors for placental villous angiogenesis and regulates fetal adrenal maturation and the onset of parturition [31], [32], [33]. During the early gestation, relatively small amounts of estrogen and progesterone are produced by the maternal ovaries. Afterward, the placenta produces huge amounts of estrogens as well as progesterone and the magnitude of hyperestrogenic state of human pregnancy is continually increasing as pregnancy progresses. Maternal plasma testosterone also increases during pregnancy and converts to E2 by aromatase activity in the placenta. The LH and FSH of pregnant women are synthesized and secreted by gonadotrophs of the anterior pituitary gland. During pregnancy, the secretion of both LH and FSH are in low level. The inhibited production of those two hormones may result from the persistently elevated levels of estrogen and progesterone [34], [35]. In this cohort, we demonstrated that there is an increase in the levels of plasma E2, progesterone, and testosterone, which is consistent with those of normal human pregnancy reported previously [36], [37], [38].

During pregnancy, estriol level increases and its concentrations will be much higher than that of E2. However, our research team is more interested in studying the association between maternal E2 level and intrauterine fetal development based on the following reasons: 1. E2 is one of the most important sex hormones during pregnancy, which influences various aspects of placental function and fetal growth. Animal experiment showed that elevated serum E2 level in the first trimester would suppress extravillous cytotrophoblast (EVT) spiral artery invasion, impair blood flow to the placenta and lead to growth restriction, which might lead to chronic diseases in later life, 2. Increasing evidences indicate that E2 plays an important role in regulating specific genes and enzymes to influence the fetal brain development, neurite growth, synaptic pattern, sex differentiation, and neonatal behavioral change, 3. Maternal high E2 level in the first trimester was found to correlate with increased risks of low birth weight (LBW) and small-for-gestational age birth (SGA), and 4. NP had the ability to mimic the effect of E2. Our previous study indicated that maternal NP exposure is associated with SGA, decreased body length at birth, and low maternal weight gain. We continue to follow-up the birth cohort and aim to explore the association between prenatal NP exposure and neurobehavioral development of early childhood. Although estriol levels increase during pregnancy and is useful in assessment of pre-term labor risk, mimic or alter the E2 levels by NP draws great concern on the developing brain [39], [40], [41].

### 4.2 Estrogenic effects of NP

The xeno-estrogen effects of NP have been reported in vivo and in vitro. Laws et al. (2000) proposed that NP had the ability to compete with E2 or promegestone by estrogen and progesterone receptors binding [10]. Sayed Ael et al. (2012) found that NP-treated Clarias gariepinus were associated with the decrease of FSH, LH and testosterone concentrations but the17-beta-estradiol level was increased [42]. Wu et al. (2010) indicated that NP has differential effects on testosterone synthesis: stimulated testosterone release through increase of both protein levels and activities of steroidogenic acute regulatory (StAR) protein and of cytochrome P450 side-chain cleavage (P450scc) protein, and in contrast, inhibited human chorionic gonadotropin-induced testosterone release in rat Leydig cells [43].

### 4.3 Estrogen negative feedback on gonadotropin-releasing hormone

In this perspective study, we demonstrated that there was a negative association between maternal plasma LH concentrations and NP level throughout the three trimesters. Considering that the diurnal changes can be observed in the levels of HCG (human chorionic gonadotropin), which is related to LH production. At Down's syndrome screening during the first trimester, maternal β-HCG was also determined. We further adjusted maternal β-HCG levels in the regression model and a significantly negative association between maternal NP and LH still existed (Table 5). Furuta et al. (2006) suggested that NP suppressed the LH secretion in adult rats by affecting the anterior pituitary [44]. The impact of similar compounds such as Bisphenol A with estrogenic effects on gonadotropin-releasing hormone mRNA expression was also reported [45]. The similar finding also indicated that Dioxin-induced LH reduction may affect gonadal steroidogenesis and cause a disorder of sex steroid biosyntheses, resulting in androgen/estrogen deficiency in the fetal brain [46], [47]. In addition, it has been proposed that estrogen exerts an inhibitory effect on the pituitary in women: negative feedback of estrogen resulted in suppressing the FSH and LH secretion [35], [48]. In this study, we found that there was a significantly negative association between maternal NP and LH, which still existed after adjusting E2 in GEE model. As an estrogen-mimic, the negative feedback of NP on LH among pregnant women could not be neglected.

**Table 5:** Multivariable regression model of LH, β-HCG, and maternal urinary NP levels in the first trimester.

	Model LH (mIU/ml)		Model^a^ LH (mIU/ml)	
Variables	β (SE)	p-value	β (SE)	p-value
NP (µg/g cre)	−0.03 (0.01)	0.04	−0.79 (0.32)	0.02
β-HCG	0.01 (0.01)	0.01	0.01 (0.01)	0.01

a : Beta of a natural log-transformation of urinary NP concentrations after adjusting covariates including age, pre-pregnancy BMI, parity and birth sex.

β = estimated coefficient.

SE = standard error of the estimated coefficient.

### 4.4 Maternal adaptations to pregnancy

The non-significant associations between NP and other hormones in this study may be attributed to the physiological adaptations of pregnant women. Physiological adaptations to pregnancy are profound, that including changes in gastric secretion and motility, changes in metabolizing enzymes in the liver, increases in cardiac output and blood volume, increases in renal blood flow and glomerular filtration, and regulations in the endocrine changes [49], [50], [51], [52]. Most of these pregnancy-induced alterations occur in response to growth and development of the fetus. The changes in toxicokinetics and toxicodynamics also occur during pregnancy by altering the activity of several hepatic cytochrome P450 enzymes and transporters in the specific stage of gestation [53]. The predominant metabolite of NP is either a glucuronide conjugate of NP or glucuronide conjugates of ring or side chain hydroxylated NP. The half-life of NP in human blood is 2–3 hours and the bioavailability of NP is 20% after oral application [54], [55]. Although the half-life of NP in the human body is short, pregnant women in this study are repetitively and persistently exposed to NP.

### 4.5 The effects of intrauterine NP exposure

The effects of intrauterine NP exposure on fetus must be considered. Kimura et al. (2006) suggest that NP exposure in utero possibly affected fetal body weight and some reproductive organ weights [56]. A dose-dependent effect of NP on decreased epididymal weight of male rats was reported [57]. Jie et al.(2010) indicated that gestational NP exposure decreased the ratio of anogenital distance to body length and influenced the learning and memory functions in offspring rats [58]. NP was proved that can cross human placenta by utilising a dual ex vivo recirculating model of placental perfusion [59]. Our previous studies also suggested that fetuses may have high NP exposure due to transplacental absorption and maternal NP levels may associate with adverse birth outcomes [27], [28], [29].

### 4.6 Maternal NP levels and dietary variation

Human NP exposure occurs mainly through the ingestion of NP-contaminated water and foods. Migration of NP from food-contact materials during food packaging or processing has also been studied [1], [5], [6], [7], [8]. It has been reported that dietary behavior, food frequency, and nutrient intake from more than 10000 pregnant women changed little during pregnancy [60]. In this study, only the frequencies of nutriment supplementation and of whole milk were significantly increased during gestation. Little variation of urinary NP concentration in the three trimesters may be attributed to the small changes in maternal dietary behavior.

### 4.7 The concentrations of NP in human from different countries

Occupationally exposed to NP includes workers in plastics, textile, detergent, and pesticide industries, etc. Our previous study indicated that urinary NP concentrations were 42.06±46.63 ng/ml after a shift and 23.50±17.34 ng/ml before a shift among textile workers. The urinary NP concentration of 79 office workers was 3.74 ng/ml [30]. The NP level in pregnant women in this study was similar to that of people without occupational NP exposure, but was higher than the levels in other countries. Calafat et al. (2005) reported that median and 95th percentile NP concentrations in 394 adult urine samples in the USA were <0.1 µg/L and 1.57 µg/L, respectively [61]. Kawaguchi et al. (2005) indicated that healthy people in Japan had urinary NP concentrations of 1.04–2.1 ng/ml [62]. In Chinese adults aged 21–29, the maximum urinary NP concentration was 2.3 ng/ml [63]. 96.9% of the pregnant women live in Taipei city. Taipei is a metropolis with few industrial activities and all pregnant women were non-occupationally exposed to NP (Table 1). The intensive use of NPEs detergents in daily activities may lead to high NP exposure levels for Taiwanese [64]. NP was detected in all rivers, sludge, and sediments in Taiwan. Previous study also reported that the average daily intake of NP for Taiwanese individuals was high in comparison with that in Germany and New Zealand [65], [66], [67].

Multiple maternal factors including age, BMI, psychological status, dietary and nutriment intake, and medication use are all associated with hormone levels during gestation [53], [68], [69], [70]. Several epidemiological studies that focused on the effects of endocrine disrupting substances have shown that toxicants exposure such as polybromodiphenyl ethers and phthalates may affect hormone levels of pregnant women and neonates [71], [72], [73]. However, these studies assessed the effects of exposure only during one stage of pregnancy. Insufficient exposure assessment may conceal the critical stage of pregnancy-induced hormone alterations during the dynamic processes of gestation.

### 4.8 Strengths and limitations

This prospective cohort design provides an informative and longitudinal evaluation of maternal NP exposure and its influence on hormone variation. The value of this study is its characterisation of NP exposure in pregnant women throughout the three trimesters. However, due to the challenge of establishing a pregnant women cohort, the relatively small sample size could be a limitation of this study. During follow-up, factors such as prenatal care, nutriment intake, and stress which may not be measured accurately need to be accounted. In addition, due to the short half-life of NP in blood, the maternal NP exposure for the three trimesters may not be representative enough of whole pregnancy. But based on the stable dietary intake pattern and repeated NP measurement in all three trimesters, the spot urine could be representative of internal NP dose during pregnancy.

Estrogenic effects of NP can be sustained among these pregnant women. This cohort study demonstrates that negative association occurs between maternal NP exposure and plasma LH levels. The estrogen-mimic effect of NP might influence the negative feedback on LH during pregnancy.
